# Chicago Medical Society

**Published:** 1883-10

**Authors:** 


### Society Reports.

ARTICLE VIII.

CHICAGO MEDICAL SOCIETY.

The society held its regular semi-monthly meeting at the Grand Pacific Hotel, on the third Monday evening of July. Dr. D. W. Graham presided.

During the session, several valuable papers were read. The first was by Dr. H. D. Valin, who read a practical paper of much scientific interest, "Mechanical Equivalent of Animal Heat." The paper forms part of a manual of biology which he is writing, and the views expressed in portions of it by the writer differ from those of older authors. As the paper has been printed in this JOURNAL in its originality, further comment thereupon is thought to be unncessary.

Dr. W. L. Axford read notes of a report of a case of bilateral and forward dislocation of the fourth cervical vertebra. The accident occurred to a litle girl eight years of age, in March of the present year, and during the past few months she has almost entirely recovered from its effects. There was no injury to the cord, nor resulting fracture. The extent of the dislocation was estimated to be two-thirds of an inch. Drs. E. Andrews and J. G. Kiernan were called to see the case, and concurred in the diagnosis. The treatment adopted was the "let alone" method. She now keeps her head more fixed and forward than the normal position is, and it may be possible that in time inflammation of the membranes of the cord may occur, although at present no trace of this can be detected, nor has any paralytic symptom been developed at any time since she was hurt.

Dr. C. T. Fenn read a report of a case of "Acute Hepatic Abscess — Mistake in Diagnosis; Free Opening; Death and Autopsy." The following synopsis is here appended:

The subject was a German lad, æt. fourteen, who had been a blacksmith's apprentice. On June 16, 1883, he went home complaining of toothache, headache, pain in the right side and cold. On the second day, his bowels moved slightly from the effects of castor oil taken the day before; he had a feverish pulse and hot skin; offensive breath; pain in his head, neck and limbs, and especially under the right breast. Tr. opii deodor., chloratis kali in glycerine and aqua distil. was given him, and emp. cantharidis 4x5 applied at night to the right side, across and above the short ribs.

On the third day, the fetor was corrected, and the pain in the side had subsided, but there remained a coated tongue, thirst, headache, tympanitis, rapid pulse, high temperature, and a tendency to cough that caused pain in his side. On the fourth day, he uttered a short cough with every breath. There was decided flatness (as Dr. F. thought) over the lower lobe of the right lung, extending from the nipple downward, and poultices were applied continuously from this time. Tympanitis was present, and a perceptible bulging of the right side. Constant resting on his back caused extreme discomfort, with perceptible shortening of the breath. On the fifth, sixth and seventh days, the temperature of the body was high; the pulse over 100 during the day, with increase at night. No tendency to spontaneous movement of the bowels; the urine was darker than normal, but skin and conjunctiva unaffected. He was obliged to sit in a chair to procure rest. In this position the heart was noticed beating between the second and fourth ribs. The dullness on the right side extended to one and a half inches above the nipple. Salicylate of soda was given him, which seemed to remove entirely the troublesome cough he had been having. There remained, however, a dark, moist coat upon the tongue, marked thirst, tympanitis and constipation, although large enemata had been introduced daily, which were retained. He loathed beef tea, but found satisfaction in tea, coffee, milk and lemonade. On the eighth, ninth, tenth and eleventh days the treatment was opiates, nutriment, expectorants and poultices. On one of those days he evacuated the bowels copiously, the contents being dark, thin, and of disagreeable odor.

On the twelfth day, he seemed much improved. The fullness in the lateral chest and abdomen disappeared. He slept and ate with evident benefit; discontinued the poultices, but had to get an easy position by sitting in his chair by day. On the fourteenth day, there was an exacerbation of symptoms; the dullness which had been lowering in his side increased; he had chills, sweats, and extreme fever, especially in the evening. On the fifteenth day, Dr. D. T. Nichols was invited to see him for the purpose of assisting in surgical interference, should the diagnosis be confirmed, which was "pleurisy with effusion." The aspirator was used, at a point about a third of the way from the median line in front to the spine, and between the eighth and ninth ribs. Pus was directly perceived. A free opening was made, followed by a copious, sluggish stream of rather offensive matter; a drainage tube was inserted, and the external wound dressed with cotton soaked in carbolized glycerine. Before closing the wound in this manner, a finger was inserted in the opening, and a smooth, soft wall, apparently held together by strong trabeculæ, was felt, and we concluded the lung was broken down at the site of the large abscess. The case seemed to be a hopeless one, considering the rapid development and quantitity of pus that escaped at the time and during the night, which was at least a pint.

On the sixteenth day, he was greatly relieved. The cavity was thoroughly cleansed with carbolized water, but it would be returned almost clear. The patient was so relieved that it seemed a work of magic had been done. The dorsal decubitus was now one of relief, and his slumbers were quiet and restful.

On the eighteenth day, the external wound was clean and dry. The washings were still continued, but septicæmia had appeared. Supposing the case to be one of abscess of the lung, we thought it strange that this was the only constitutional symptom present, although he had a return of the chills, followed by extreme heat, and cleansing the interior was on the whole unsatisfactory the three subsequent days. While deliberating what new means we would devise, the patient had a rigor from which he did not rally, and died on the morning of the twenty-second day, notwithstanding the free administration of quinine, other tonics, etc. etc., that had been given him in addition to the various other remedies.

*Autopsy*—twelve hours after death; body surrounded by ice, distended and purplish-yellow appearance of face, neck, chest and abdomen, as if decomposition were quite advanced. Incision from top of sternum to three inches below umbilicus; contents of chest exposed. The lungs were both healthy, and crowded up to occupy half their normal space. The heart was beneath the second and third ribs. The liver extended across the body on a line above the level of the nipples. The stomach and intestines distended, aided in forcing it high. The opening to the abscess was directly over the thickest portion of the liver, and about one-third of the organ seemed to be involved. The peritonæum was not inflamed; the spleen was enlarged and softened. No other observations were made, and it was not easy to determine how the abscess had its origin.

Dr. W. H. Curtis asked the author of the paper if diarrhœa had been present any time preceding his sickness. Answered, negatively.

Dr. D. R. Brower inquired if jaundice was present. Answered, no; excepting shortly after death when the surface turned yellow.

Dr. Axford inquired if the edges of the liver became adherent to the walls of the abdomen. Answered, no.

Dr. Valin had seen a case of abscess of the liver during the spring of last year. The patient (a man) had malaria twelve years previously. The inflammation lasted nine to eleven days, when the abscess was opened and eight ounces of pus removed. There was no jaundice nor diarrhœa in the case, but decided motion of the heart was noticed.

Dr. L. H. Montgomery asked Dr. Fenn what different method of treatment would have been pursued had they diagnosed the case to be an abscess of the liver? Answered, the treatment would have been substantially the same, and this was one reason why he reported the case, as well as the somewhat comparative infrequency of this trouble in private practice.

Dr. Curtis then recited briefly the histories of four cases of hepatic abscess that he had met with in the temperate zone. The first occurred in an Indian boy, and pointed toward the ribs. A free incision was made, and there was evacuated a mass of lumbricoid worms as large as a fist, followed by a discharge of pus. In this case the worms caused the abscess. The boy recovered.

The second case occurred in a habitual drunkard who had chronic liver trouble. During one of his periodical sprees he was exposed to cold and dampness resulting in an abscess. Adhesions formed ; an opening was made and a large quantity of pus discharged. He, too, recovered.

The third case was an old lady sixty-two years old. She also had marked jaundice, and marked symptoms of septicæmia. As purpura set in, in two weeks it ruptured into the lung, when she expectorated a good deal of pus. She rallied and got up. Then she was attacked with chills and fever, and became "hectic." It "gave way" again, this time into the stomach, so that she vomited the pus, but she finally made a good recovery. She had a cicatrix in her side since sixteen years of age, when a number of gall stones had been removed, over forty years ago.

The fourth case was that of a railroad president who had been under Dr. C's care for a fortnight. He diagnosed the trouble to be abscess of the liver, and wished to aspirate it. The friends of the patient desired counsel in New York, and he was accordingly taken there, when Dr. Hammond aspirated him, followed by a good recovery.

Dr. D. R. Brower exhibited the heart of a patient that had died of endocarditis, in which there was an extensive lesion and roughening of the mitral valve. The trouble dated from an attack of puerperal fever of a year ago. No possible abnormal condition of the heart could be detected. There had been no murmur present, and Dr. B. thought the valve was therefore perfectly sufficient in its action. No murmur could be detected after exercise. The patient had shortening of breath. She was anæmic and greatly emaciated. An embolism was also found. The rarity of the case when such serious lesions were present, was his reason for presenting it before the society. She also had chorea ; and to him the case and pathological specimen had elicited much interest. The society then adjourned *sine die*. L. H. M.

